# Noncoding RNAs regulating ferroptosis in cardiovascular diseases: novel roles and therapeutic strategies

**DOI:** 10.1007/s11010-023-04895-w

**Published:** 2023-12-08

**Authors:** Changyong Wu, Suli Bao, Huang Sun, Xiaocui Chen, Lu Yang, Ruijie Li, Yunzhu Peng

**Affiliations:** 1 Department of Cardiology, the First Affiliated Hospital of Kunming Medical University, Kunming, China; 2 Department of Gastroenterology, Affiliated Hospital of Panzhihua University, Panzhihua, China

**Keywords:** Noncoding RNA, Ferroptosis, Cardiovascular diseases, Iron metabolism, Lipid peroxidation, Molecular mechanism

## Abstract

The morbidity and mortality rates of cardiovascular diseases (CVDs) are increasing; thus, they impose substantial health and economic burdens worldwide, and effective interventions are needed for immediate resolution of this issue. Recent studies have suggested that noncoding RNAs (ncRNAs) play critical roles in the occurrence and development of CVDs and are potential therapeutic targets and novel biomarkers for these diseases. Newly discovered modes of cell death, including necroptosis, pyroptosis, apoptosis, autophagy-dependent cell death and ferroptosis, also play key roles in CVD progression. However, ferroptosis, which differs from the other aforementioned forms of regulated cell death in terms of cell morphology, biochemistry and inhereditability, is a unique iron-dependent mode of nonapoptotic cell death induced by abnormal iron metabolism and excessive accumulation of iron-dependent lipid peroxides and reactive oxygen species (ROS). Increasing evidence has confirmed that ncRNA-mediated ferroptosis is involved in regulating tissue homeostasis and CVD-related pathophysiological conditions, such as cardiac ischemia/reperfusion (I/R) injury, myocardial infarction (MI), atrial fibrillation (AF), cardiomyopathy and heart failure (HF). In this review, we summarize the underlying mechanism of ferroptosis, discuss the pathophysiological effects of ncRNA-mediated ferroptosis in CVDs and provide ideas for effective therapeutic strategies.

## Introduction

CVDs remain major causes of death and disability in developing and developed countries [1, 2]; these diseases mainly include atherosclerosis, coronary artery diseases, myocardial I/R injury, hypertension, arrhythmia, heart valve disorders, cardiomyopathy and HF [3]. However, the heart is primarily composed of terminally differentiated cardiomyocytes that have limited regenerative capacity and physiological significance in regulating cardiac development, senescence and homeostasis [4, 5]. Therefore, a clear understanding of cardiovascular pathophysiology is need for the prevention, diagnosis, treatment and prognosis of CVDs.

A myriad of studies have indicated that cell death plays a central role in regulating tissue and cellular homeostasis and the pathogeneses of various diseases. Fundamentally, cell death is classified into two different types: accidental cell death (ACD) and regulated cell death (RCD) [6]. ACD is a passive process in which severe damage to cells is incurred via stimulation by various pathogenic factors. However, RCD, also known as programmed cell death, is an active process under physiological conditions and can be further classified into apoptotic and nonapoptotic forms. Many nonapoptotic forms of RCD, such as necroptosis, pyroptosis, apoptosis, autophagy-dependent cell death and ferroptosis, have been identified, and they exert a significant influence on the occurrence and progression of numerous diseases, particularly CVDs [6, 7]. Additionally, recent studies have identified other RCD modes, such as cuproptosis [8, 9] and PANoptosis [10, 11]. However, ferroptosis is the most recently discovered type of RCD. In 2012, Dixon et al. [12] first proposed ferroptosis as a unique iron-dependent form of nonapoptotic cell death that is triggered by the oncogenic RNA-selective lethal (RSL) small-molecule erastin in rat RAS-mutant tumor cells. Ferroptosis, which is distinct from other forms of RCD in terms of morphology, biochemistry, genetics and metabolic manifestations, is dependent on the intracellular iron level but not the level of other metals [7, 12]. Intriguingly, ferroptotic cell death is associated with significant abnormal changes in mitochondrial morphology, including shrinkage, swelling, a reduction in the number or disappearance of cristae, a decrease in the membrane density and rupture of the outer membranes, while the morphology of the nucleus remains unchanged [13, 14].

Studies have shown that ferroptosis is extensively involved in the pathophysiology of various conditions, such as neurodegenerative diseases [15], cancer [16], acute kidney injury [17], liver disease [18] and I/R injury [19], and it is closely related to iron, lipid and antioxidant metabolism pathway impairment. Moreover, recent studies have uncovered links between ferroptosis and CVDs. For example, histochrome treatment decreases intracellular and mitochondrial ROS levels which rescues the myocardium from I/R injury by preventing ferroptotic cell death [20]. Therefore, insight into the mechanism underlying cardiac cell ferroptosis is crucial for obtaining an in-depth understanding of the pathogenesis of CVDs.

ncRNAs, a class of RNA molecules that do not encode proteins, are generally classified into two categories based on their nucleotide length: short or small-chain ncRNAs (including microRNAs, miRNAs) comprising approximately 18–200 nucleotides and long-chain ncRNAs (lncRNAs), comprising more than 200 nucleotides [21, 22]. In addition, lncRNAs can be further classified into six types according to their relative position to neighboring coding regions in the genome: sense lncRNAs, antisense lncRNAs, intergenic lncRNAs, intronic lncRNAs, enhancer lncRNAs and bidirectional lncRNAs [23]. In addition to linear ncRNAs, nonlinear ncRNAs, called circular RNAs (circRNAs), has been identified; circRNAs can be classified into three categories depending on their source: intronic circRNAs (circular intronic RNAs, ciRNAs), exonic circRNAs (exonic circular RNAs, ecRNAs), and exonic and intronic circRNAs (exon_intron circular RNAs, EIciRNAs) [24–26]. ncRNAs can function either alone or with other ncRNAs by acting as competitive endogenous RNAs (ceRNAs) to sponge miRNAs, which prevents them from interacting with target genes or proteins. In recent years, a large number of studies have demonstrated that both ncRNAs and ferroptosis are involved in the occurrence and progression of various CVDs and that ncRNAs regulate ferroptosis through key factors, such as SLC7A11, TFR, GPX4 and ACSL4. However, further analysis is needed to identify the pathophysiological mechanisms of CVDs that are affected by the regulatory actions of ncRNAs on ferroptosis.

In this review, we summarize the ferroptosis-related signaling pathways and discuss recent developments related to and perspectives on the role of ncRNA-regulated ferroptosis in CVD pathophysiology to identify potential therapeutic strategies for CVDs.

## Ferroptosis signaling pathways and their roles in CVDs

**Fig. 1:**
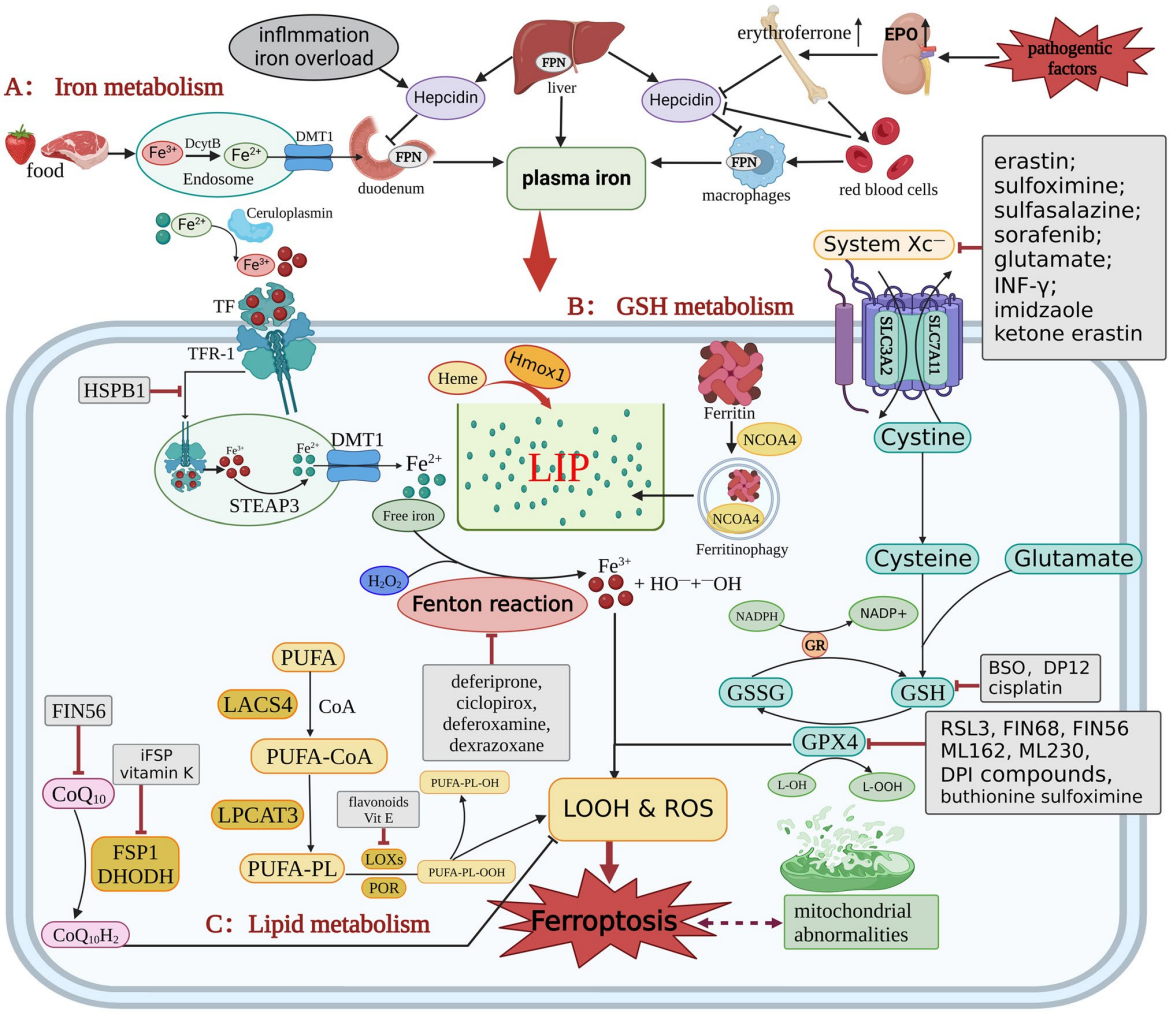
Molecular signaling mechanisms and pathways involved in ferroptosis. Different physiological processes and pathological stresses may trigger ferroptosis. Abnormal iron metabolism, excess ROS production and excessive accumulation of lipid peroxide are important factors that induce ferroptosis. Abbreviations: DcytB: duodenal cytochrome B reductase; EPO: erythropoietin; GSSG: glutathione disulfide; iFSP: inhibitor of FSP1; L-OOH: lipid peroxide; L-OH: lipid alcohol; and NADPH: nicotinamide adenine dinucleotide phosphate

In recent years, significant achievements have been made in elucidating the molecular mechanism of ferroptosis. Ferroptosis is defined as a newly discovered form of RCD that involves various processes and metabolic pathways, including iron homeostasis, amino acid, lipid metabolism and the antioxidant system (Fig. 1). However, the regulatory mechanisms of ferroptosis are intricate. Accumulating experimental evidence indicates that ferroptosis has a vital role in the pathogenesis of various CVDs, including atherosclerosis, MI, I/R, AF, cardiomyopathy, cardiac hypertrophy and HF [27]. Studies have shown that ferroptosis contributes to the occurrence and development of CVDs by impairing the structure and function of the heart and blood vessels and disrupting their normal function [28]. However, the precise underlying mechanisms of various CVDs are not completely clear. Fully elucidating the association of ferroptosis with CVD pathogenesis will provide novel insights for developing effective therapeutic strategies for patients with CVDs.

## Iron metabolism and homeostasis

Iron is an essential trace element in the human body due to its participation in the synthesis of many proteins and enzymes [29]. Iron is stored in hemoglobin (72%), myoglobin (3%), and other compounds (0.2%), reserve iron (25%) is stored in ferritin in the liver, spleen and bone marrow [30]. Iron is involved in various metabolic processes and life activities, and it is an indispensable cofactor for certain enzymes required for oxygen transport, cell respiration and electron transfer, energy metabolism, DNA synthesis and immune regulation [31, 32]. The complex and precise mechanisms of iron homeostasis regulation ensure that the iron concentration in a cell remains stable and prevent intracellular iron overload from triggering the Fenton reaction, which in turn leads to ferroptosis [13] (Fig. 1A). The main source of iron in the body is hemoglobin, and iron is recovered from hemoglobin by macrophages that phagocytose senescent red blood cells. Simultaneously, the duodenum absorbs dietary iron ions in the human body. However, chronic inflammation and HF-related generalized edema reduce iron absorption in gastrointestinal system and macrophage phagocytosis of red blood cells, resulting in functional iron deficiency which is associated with HF, cerebrovascular diseases, AF and pulmonary hypertension [33]. The effects of iron supplementation have been evaluated in patients with HF, and it has been reported that iron supplementation can improve quality of life and reduce hospitalization [34].

Iron obtained through these two pathways is transported in the plasma by ferroportin (FPN), also known as SLC40A1, which is widely distributed on the surface of duodenal epithelial cells, macrophages and hepatocytes. FPN function is regulated by hepcidin, a 25-amino acid liver -secreted peptide hormone that binds to FPN to promote FPN internalization and degradation when the iron concentration is high [30, 35]. However, overexpression of miR-124 inhibits the expression of FPN, leading to intracellular iron accumulation and triggering ferroptosis [36]. Bao and colleagues [37] showed that cigarette tar caused severe iron overload and lipid peroxidation, upregulated hepcidin expression and downregulated FPN and SLC7A11 in macrophages, resulting in the promotion of atherosclerosis progression. These regulatory changes can be reversed by ferroptosis inhibitors or hepcidin knockdown, while NF-κB inhibitors have the opposite effect and then inhibit macrophage ferroptosis.

The iron metabolism pathway is necessary for the intracellular accumulation of lipid peroxides and ferroptosis. Ferrous iron (Fe^2+^) in the extracellular space can be oxidized into ferric iron (Fe^3+^) by ceruloplasmin or hephaestin on the basolateral side of a cell, and Fe^3+^ is endocytosed into the cell by binding to transferrin (TF) and the membrane protein TF receptor 1 (TFR1) [38]. Within a cell, Fe^3+^ is located in endosomes and then reduced to Fe^2+^ by the iron oxide reductase six-transmembrane epithelial antigen of prostate 3 (STEAP3) and ultimately transported from endosomes to the cytosol by divalent metal transporter 1 (DMT1). Intracellular Fe^2+^ is stored in unstable labile iron pools (LIPs), and iron stored in ferritin is reduced to Fe^2+^ and consumed during the synthesis of iron-dependent enzymes [39]. Moreover, iron input is increased by iron transporter 1 (FPN1), poly copper-iron oxidase (e.g., ceruloplasmin) and ion transporter lipocalin 2 (LCN2). Once the balance between iron absorption, utilization and recycling is disrupted, cells can undergo iron overload. Iron in unstable LIPs, especially that obtained by the ferrous iron-induced Fenton reaction in the presence of hydrogen peroxide (H_2_O_2_), contributes to lipid peroxidation and ROS generation, ultimately triggering ferroptosis.

Recent studies have shown that multiple regulators of iron metabolism are involved in ferroptosis. For example, nuclear receptor coactivator 4 (NCOA4), a cargo receptor involved in ferritin autophagy, maintains intracellular and systemic iron homeostasis by delivering ferritin to lysosomes and degrading ferritin via selective cargo-mediated autophagy (a specific form of autophagy called ferritinophagy). Inhibition of ferritinophagy or knockdown of NCOA4 effectively ameliorates cellular or mitochondrial iron overload, lipid peroxide production and ROS accumulation, which protects cardiomyocytes from ferritinophagy-mediated ferroptosis and alleviates myocardial inflammation and fibrosis [40–42]. Heat shock protein beta 1 (HSPB1), a member of the small heat shock protein family, acts as a negative regulator of ferroptosis by inhibiting iron intake and the production of lipids. HSPB1 phosphorylation, regulated by protein kinase C, reduces the rate of iron-dependent production of lipid ROS to resist ferroptosis [43]. Emerging evidence indicates that overexpression of HSBP1 can accelerate recovery from tachypacing-induced structural damage and contractile dysfunction in HL-1 cardiomyocytes. Furthermore, HSBP1 can shield myofibrils from degradation by binding to them and stabilize sarcomeric proteins, indicating that HSBP1 induction is a potential target to reverse AF-induced cardiac remodeling [44]. In contrast, iron regulatory protein (IRP) and hypoxia-inducible factor-1 (HIF-1) increase cellular iron uptake by elevating TFR1 expression, thereby increasing cell sensitivity to ferroptosis [45–47]. Heme oxygenase 1 (Hmox1) is an essential enzyme in heme catabolism. Mechanistically, upregulation of Hmox1 promotes heme degradation and the release of free iron, and free iron accumulates in mitochondria and triggers lipid peroxidation, ultimately resulting in ferroptosis and myocardial injury in the presence of doxorubicin (DOX) [48, 49].

However, in recent years, a series of key regulators that pharmacologically inhibit ferroptosis have been identified. Most ferroptosis inhibitors include iron chelators and lipophilic RTAs. Iron chelators, such as deferoxamine (DFO), dexrazoxane (DXZ), deferiprone and ciclopirox, can reduce labile iron levels and block the Fenton chain reaction, thereby preventing the activation and spreading of lipid peroxidation to protect against ferroptosis [50, 51]. DXZ is the only FDA-approved drug for preventing DOX-induced cardiotoxicity in patients with cancer; DXZ can cross the cell membrane, directly enter mitochondria in cardiomyocytes and chelate intracellular free iron [52, 53].

## GSH metabolism

Glutathione peroxidases (GPxs) plays a predominant role in a variety of human diseases by decreasing hydroperoxide levels. A substantial body of evidence has shown that the GPx family consists of eight members: cytosolic GPX (cGPX, GPX1), plasma GPX (pGPX, GPX3), gastrointestinal GPx (GI‐GPx, GPX2), GPX6, and phospholipid hydroperoxide GPx (PHGPX, GPX4) [54, 55]. GPX4, a monomeric protein, is expressed as three isoforms: cytosolic (cGPX4), mitochondrial (mGPX4) and sperm nuclear GPX4 (snGPX4) [56–58]. GPX4 plays an indispensable role in maintaining membrane lipid homeostasis by preventing excess accumulation of toxic lipid peroxides and the formation of free radicals, thereby attenuating ferroptosis (Fig. 1B). Notably, these outcomes can be driven by the direct inhibition of GPX4 and by inhibition of glutathione (GSH) synthesis which involves cysteine, glutamate and glycine and proceeds in two steps. System Xc^−^, a cystine-glutamate transport receptor, is composed of two proteins, 12 channel transmembrane protein transporter solute vector family 7 member 11 (SLC7A11) and single-channel transmembrane regulatory protein solute vector family 3 members 2 (SLC3A2), which are expressed on the cell membrane and capable of maintaining redox homeostasis [59]. Extracellular cystine is exchanged for intracellular glutamate at an equal ratio by System Xc^−^. Regardless of the pathway, ultimately, an increase in GPX levels reduces the levels of lipid-based ROS, which increase unsaturated fatty acid levels on the cell membrane and ultimately cause liposome peroxidation and ferroptosis. Studies have shown that DOX and sorafenib can decrease the levels of GPX and SLC7A11 and accelerate ferroptosis [60, 61]. As a result, targeting ferroptosis might be a novel therapeutic approach for preventing DOX- or sorafenib-induced cardiotoxicity in the future.

However, the high extracellular concentrations of glutamate and other molecules, such as erastin, sulfoximine, sulfasalazine and sorafenib, deplete intracellular cystine content by suppressing the function of System Xc^−^, which activates GPX4 by depleting GSH and initiating ferroptosis [62, 63]. Additionally, treating cells with direct inhibitors of GPX4, such as RSL3, ML162, FIN56, DPI 10, DPI12, DPI13 or DPI7, initiates ferroptosis by inactivating GPX4 [62, 64]. Certain synthetic compounds can suppress GPX4 and increase the peroxide level in cardiomyocytes.

## Lipid peroxidation

The lipid bilayer of the cell membrane is essential for maintaining membrane function. Cell membranes are composed mainly of lipids, proteins and carbohydrates, polyunsaturated fatty acids (PUFAs) are important components of the cell membrane [65]. The location and content of PUFAs can be used to assess the degree of ferroptosis because this mode of cell death affects the extent of intracellular lipid peroxidation. PUFAs are the lipids most susceptible to peroxidation during ferroptosis. Lipoxygenases and phosphorylase kinase G2 are two key regulators of lipid peroxidation during ferroptosis [66, 67]. PUFA-containing phospholipids (PLs) are the major substrates in ferroptotic lipid peroxidation, and PL hydroperoxides, including phosphatidylcholine, cardiolipin and phosphatidylethanolamine (PE), are involved in ferroptosis [68, 69].

Two key lipid metabolism-associated enzymes, acyl-CoA synthase long-chain family member 4 (ACSL4) and lysophosphatidylcholine acyltransferase 3 (LPCAT3), are involved in the synthesis of PUFA-PLs [70]. ACSL4 catalyzes the binding of PUFAs and adrenergic acid to coenzyme A (CoA) to generate PUFA-CoA, and then LPCAT3 catalyzes the incorporation of PUFAs from PUFA-CoA into membrane PLs to generate PUFA-PLs, resulting in the remodeling of membrane phospholipids and influencing ferroptosis [71]. ACSL4 catalyzes PUFA-CoA formation to influence ferroptosis and is a marker of ferroptotic cell death sensitivity [47, 72, 73]. ACSL4 knockout decreases PUFA-PL generation, decreasing the percentage of cell undergoing RSL3-induced ferroptosis. Knockout of ACSL4 exerts a cardioprotective effect by decreasing iron accumulation, oxidative stress and ferroptosis after myocardial ischemia in rat [74]. In addition, PUFA-PL is oxidized by lipoxygenases (LOXs) or cytochrome P450 oxidoreductase (POR) into the harmful lipid peroxidation products PUFA-PL-OH and PUFA-PL-OOH, respectively, which induce lipid peroxidation and ROS production [75–77]. Ferroptosis is activated by cellular lipid hydroperoxides through LOXs and autoxidized peroxyl radicals. Therefore, inhibiting LOX enzyme activity with flavonoids and members of the vitamin E family can reduce cell damage caused by ferroptosis [68]. Moreover, the level of the aforementioned harmful substances can be reduced by GPX4, which converts them into nontoxic lipid alcohols and water. From the opposite point of view, reducing GPX4 levels via GPX4 treatment with inhibitors such as RSL3 can promote the excessive accumulation of peroxide products and thus induce ferroptosis [78, 79] (Fig. 1C).

## Other signaling pathways

The FSP1-CoQ-NAD(P)H axis, which protects the myocardium in CVDs, is important in the GSH and GPX4 pathways. CoQ10 is mainly found in two forms, ubiquinone (the oxidized form) and ubiquinol (the reduced form); among these two forms, ubiquinol plays the main role in inhibiting oxidation. CoQ10 is a lipophilic electron carrier that participates in the mitochondrial respiratory chain and is a lipophilic radical scavenger at the plasma membrane [80]. Randomized controlled trials and meta-analyses have suggested that CoQ10 can improve cardiac function by reducing systemic oxidative stress, which is associated with a reduction in hospitalizations and the risk of cardiovascular mortality [81, 82]. Moreover, ferroptosis suppressor protein 1 (FSP1), also known as p53-responsive gene3 (PRG3), binds membranous structures in cells such as the Golgi apparatus, plasma membrane and perinuclear structures. Studies have found that FSP1 is a key component of nonmitochondrial CoQ10 and is recruited to the plasma membrane through the myristoylation of specific N-terminal sequences to capture lipotropic free radicals and exert antioxidant effects [83, 84], thereby mediating NADH-dependent CoQ reduction, suppressing CoQ activity in cells, and eventually inhibiting phospholipid peroxidation and ferroptosis via the FSP1-CoQ-NAD(P)H axis. Ara et al. [85] found that iFSP, an inhibitor of FSP1, can be used to eliminate lung cancer cells and induce ferroptosis. Upregulating FSP1 was found to alleviate septicemia-induced myocardial injury by inhibiting ferroptosis in vivo [86]. The combination of GPX4 and FSP1 serves as a separate defense system to suppress lipid peroxidation in the cytoplasmic and mitochondrial membranes, effectively alleviating CVDs by defending against ferroptosis [87]. CoQ10H2, the product of CoQ10 reduction, suppressed adipocyte differentiation and inhibited lipid ROS accumulation. In summary, elucidating the mechanism by which ferroptosis is regulated may provide new insights for the development of strategies to prevent and treat CVDs.

## Noncoding RNAs that regulate ferroptosis in CVDs

To date, some ncRNAs have been found to play potentially critical roles in CVDs by directly or indirectly regulating iron metabolism and ferroptosis-related signaling pathways. Cardiac modeling, manifesting as abnormal deposition of collagen and dysregulated extracellular matrix homeostasis, is a common, persistent and irreversible pathological change associated with CVDs and results in cardiac structural changes, cardiac dysfunction, HF and even sudden death. Therefore, one strategy to prevent adverse cardiac remodeling and consequent cardiovascular events is to go upstream and decrease cardiac cell death. Recent reports suggest that ferroptosis is involved in the initial myocardial cell death caused by MI, I/R injury, AF, cardiac hypertrophy, aortic valve replacement, cardiomyopathy and HF [88]. For instance, inhibition or knockout of xCT can exacerbate cardiomyocyte hypertrophy and angiotensin II (Ang II)-induced cardiac fibrosis in mice and increase the levels of the ferroptosis biomarkers Ptgs2, MDA and ROD, while Fer-1 can alleviate the exacerbation of cardiac hypertrophy caused by inhibiting xCT in rat cells or ablating xCT in mice [89]. In addition, GPX4 also plays an important role in promoting the thrombospondin 1 (TSP1)/autophagy, insulin-like growth factor 1 (IGF1) or stimulator of interferon genes (STING) axis to inhibit cardiac fibrosis and the expression of the associated markers α-SMA, collagen type I (Col I) and Col III [55]. In this section, we describe the pathological roles of ferroptosis-related ncRNAs in CVDs (Tables 1 and 2).

**Table 1:** MiRNAs implicated in ferroptosis regulation in CVDs

MiRNA and expression	Disease	Experimental phenotype	Target and expression	Repression/induction of ferroptosis	Cardiac fibrosis	References
miR-15a-5p↑	AMI	C57BL/6 J mice HL-1 myocardial cells	GPX4↓	Induction	–	[97]
miR-23a-3p	AMI	C57BL/6 J mice cardiomyocyte	DMT1^*^↓	Repression	–	[98]
	AF	beagle H9C2 myocardial cells	SLC7A11↑	Induction	Anti	[111]
miR-30d↓	MI	SD rat H9C2 myocardial cells	ATG5^*^↓ GPX4↑	Induction	Pro	[99]
miR-375-3p↑	MI	SD rat CF cell model	GPX4↓, ROS↑, SOD↓	Induction	Pro	[93]
miR-29b-3p↑	I/R	C57BL/6 J mice H9C2 myocardial cells	PTX3^*^↓	–	Pro	[102]
miR-135b-3p↑	I/R	SD rat H9C2 myocardial cells	GPX4^*, #^↓	Induction	Pro	[101]
miR-190a-5p↓	I/R	H9C2 myocardial cells	GSL2^*^↑	Repression	Anti	[103]
miR-196c-3p	I/R	SD rat H9C2 myocardial cells	NOX4↓, P53↓, LOX↓	Repression	Anti	[105]
miR-199a-5p↑	I/R	H9C2 myocardial cells	Akt/eNOS^*^↓	Induction	Pro	[104]
miR-210-3p↑	I/R	H9C2 myocardial cells	TFR^*^↓	Repression	Anti	[106]
miR-1224↑	I/R	H9C2 myocardial cells	GPX4^*^↓	Induction	–	[100]
miR-143-3p↑	AF	C57BL/6 J mice cardiomyocyte	GOT1^*^↓	Repression	–	[112]
miR-351↓	HF	C57BL/6 J mice	MLK3^#^↑	Repression	Anti	[107]

↑ enhanced effect, ↓ reduced effect,—the effect is not different; *cell transfection, ^#^gene mouse mode

**Table 2:** LncRNAs and circRNAs implicated in ferroptosis regulation in CVDs

LncRNA, circRNA and expression	Disease	Experimental phenotype	Target and expression	Repression/induction of ferroptosis	Cardiac fibrosis	References
lncRNA AABR07025387.1↑	I/R	SD rat H9C2 myocardial cells	miR-205^#^↓, ACSL4↑	Induction	Pro	[114]
Exo-lncRNA Mir9-3hg↑	I/R	C57BL/6 J mice HL-1 myocardial cells	Pum2^*^↓, PRDX6↑	Repression	Anti	[115]
lncRNA Gm47283↓	MI	C57BL/6 J mice HL-1 myocardial cells	miR-706^#^↑, Ptgs2↓	Repression	Anti	[116]
lncRNA AABR07017145.1↑	Cardiac hypertrophy	Wistar rat CMEC	miR-30b-5p↓, MMP9/TIMP1^*, #^↓, TFR-1↑	Induction	Pro	[120]
lncRNA ZFAS1↓	DCM	C57BL/6 J mice cardiomyocyte	miR-150-5p^*, #^↑, CCND2^*, #^↑	Repression	Anti	[117]
lncRNA KCNQ1OT1↑	DOX	SD rat AC16 cardiomyocyte	miR-7-5p↓	Induction	Pro	[121]
lncRNA SEMA5A-1T1↑	Cardiac valve disease	patient AC16 cardiomyocyte	miR-143-3p↓, BCL2^*^↑ SLC7A11^*^↑	Repression	Anti	[126]
circ RNA1615↓	MI	C57BL/6 J mice HL-1 myocardial cells	miR-152-3p↑, LRP6^*^↓	Repression	Anti	[127]
circ Snx12↓	HF	C57BL/6 J mice HL-1 myocardial cells	miR-224-5P↑, FTH1↓	Induction	Pro	[128]
circ FEACR↑	I/R	C57BL/6 J mice H/R-cardiomyocytes	NAMPT^#^↑, Sirt1↑, FOXO1↓, FTH1^#^↓	Repression	Anti	[129]
circ Cmss1↑	Cardiac hypertrophy	C57BL/6 J mice	EIF4A3↑, TFR1^#^↑	Induction	Anti	[130]

↑ enhanced effect, ↓ reduced effect; *cell transfection, ^#^gene mouse model

## MiRNAs that regulate ferroptosis in CVDs

MiRNAs are a ubiquitous class of endogenous noncoding small RNA molecules, and there are many types of miRNAs in the body. They are generally 18–25 nucleotides in length and bind the 3’ untranslated region (UTR) of target mRNAs to regulate the expression of different genes by interfering with mRNA molecules at the posttranscriptional and translational levels. In recent years, the detailed molecular mechanisms underlying ncRNA-mediated ferroptosis in cancer have been explored [90, 91], but the function of ferroptosis-related miRNAs in CVDs remains largely unknown.

Acute MI (AMI) causes a series of pathological responses in cardiomyocytes, including ischemia, hypoxia, inflammatory reactions and necrosis in the early stage and persistent ventricular remodeling, ischemic cardiomyopathy, HF and even death in the late stage [92, 93]. Early restoration of vascular perfusion, such as through percutaneous coronary intervention (PCI) or thrombolytic therapy, can reduce the number of necrotic cardiomyocytes and the risk of adverse cardiovascular events. However, cardiomyocyte damage and dysfunction persist even after reperfusion of myocardial tissue, and reperfusion inducing myocardial necrosis and the development of cardiac fibrosis; these changes are collectively referred to as I/R injury [94]. Recent studies have revealed that iron homeostasis and miRNAs play indispensable roles in maintaining cardiac structure and function [68, 95, 96]. Either iron deficiency or iron overload can impair cardiac function. Fan et al. [97] verified that inhibition of miR-15a-5p by silencing the transcription factor early growth response-1 (Egr-1) can suppress ferroptosis and alleviate myocardial injury by increasing GPX4 and MDA levels and decreasing the activity of ROS in an AMI mouse model. MiR-23a-3p from the human umbilical cord blood (HUCB)-mesenchymal stem cell (MSC)-derived exosomes targets DMT1 to inhibit ferroptosis and attenuate myocardial injury [98]. Tang et al. [99] confirmed that targeting ATG5 by overexpression of miR-30d effectively inhibits autophagy upregulates FTH1 and GPX4 expression in H9C2 cells, and increases the content of GSH, protecting against ferroptosis-mediated myocardial injury. Furthermore, miR-375-3p expression is significantly upregulated in an I/R rat model, and miR-375-3p targets GPX4-related ferroptosis to promote cardiac fibrosis, which is associated with increased collagen I expression and ROS levels and inhibition of the oxidative scavenging by superoxide dismutase (SOD). However, this process was found to be reversed by a miR-375-3p inhibitor and the ferroptosis inhibitor ferrostatin-1 (Fer-1) [93].

As mentioned above, GPX4 acts as an important mediator of I/R injury in cardiac tissue by regulating ferroptosis and alleviating oxidative damage via the elimination of lipid peroxides from various membranes. Inhibition of miR-1224 and miR-135b-3p can alleviate myocardial injury by targeting GPX4 and suppressing hypoxia/reoxygenation (H/R)-induced oxidative stress [100, 101]. However, increased expression of miR-135b-3p exacerbates myocardial I/R injury in an iron-dependent manner in vitro [101]. Similarly, miR-29b-3p also aggravates cardiomyocyte injury and promotes the secretion of inflammatory cytokines such as TNF-α caused by I/R through targeted downregulation of pentraxin 3 (PTX3) [102]. Additionally, other miRNAs, such as miR-190a-5p, miR-199a-5p, miR-196c-3p and miR-210-3p play critical roles in myocardial I/R injury by regulating ferroptosis-related genes and proteins [103–106]. In the later stage of MI, MI will cause left ventricular enlargement and cardiac fibrosis progression occur, ultimately leading to HF. Ji et al. [105] found that an miR-196c-3p mimic improves cardiac function by inhibiting the expression of the ferroptosis hub genes NOX4, p53 and LOX in a myocardial I/R injury mouse model. Wang et al. [107] showed that a miR-351 agomir effectively inhibited mixed lineage kinase 3 (MLK3) protein and mRNA expression to regulate JNK/p53 signaling pathway-mediated oxidative stress, which significantly increased the left ventricular ejection fraction (LVEF) and left ventricular fractional shortening (LVFS), decreased the left ventricular end-diastolic diameter (LVEDD), the left ventricular end-systolic diameter (LVESD), LV mass and collagen deposition, improved cardiac function, and inhibited cardiac hypertrophy and fibrosis in the advanced stage of congestive heart failure (CHF).

AF, mainly characterized by persistent biventricular enlargement and cardiac diastolic dysfunction, is the most common arrhythmia. Recent reports have revealed that ncRNAs play significant roles in the pathophysiological mechanism of AF [108–110]. However, the molecular mechanisms underlying AF and ferroptosis remain to be fully elucidated. Liu et al. [111] suggested that inhibition of cardiac fibroblast-derived exon-miR-23a-3p upregulates SLC7A11 and protects H9C2 cardiomyocytes from ferroptosis, preventing persistent AF development. In addition, miR-143-3p overexpression in AF cardiomyocytes was found to increase cell proliferation and viability, inhibit lipid ROS production and mitochondrial superoxide formation and reduce the intracellular concentrations of total iron and Fe^2+^ by inhibiting glutamic-oxaloacetic transaminase 1 (GOT1)-mediated oxidative damage and cardiac ferroptosis [112]. As indicated above, miRNAs play key roles in CVDs by regulating ferroptosis-related signaling pathways, as shown in Table 1.

## LncRNAs that regulate ferroptosis in CVDs

LncRNAs are transcripts exceeding 200 nucleotides in length that regulate different cellular pathways and the expression of genes by binding to DNA, mRNA, miRNA and protein. LncRNAs play important roles in regulating physiological and pathological processes in multiple CVDs mainly through cis- or trans-regulation of chromosome structure, translation, alternative splicing at the transcriptional and posttranscriptional levels, and mRNA transport, stability and translation [113]. Based on the current research evidence, we believe that lncRNAs play vital roles in CVDs, especially MI, by acting as ceRNAs and mediating cardiomyocyte ferroptosis (Table 2). For instance, silencing lncAABR07025387.1 can attenuate myocardial I/R injury by increasing cardiomyocyte activity and decreasing lipid ROS, Fe^2+^, ACSL4 and LPCAT3 levels, which can suppress ACSL4-mediated ferroptosis. In addition, lncAABR07025387.1 serves as a ceRNA to sponge miR-205 which directly targets ACSL4. Moreover, downregulation of miR-205 efficiently reverses the inhibition of ACSL4-mediated ferroptosis [114]. Zhang and colleagues found that the exo-lncRNA Mir9-3hg binds and downregulates pumilio RNA-binding family member 2 (Pum2). Pum2 is ribonucleic acid-binding protein (RBP) that inhibits peroxiredoxin 6 (PRDX6) protein expression by binding to the PRDX6 promoter region, so its downregulation facilitates cell proliferation, increases the GSH content, and reduces the iron ion concentrations, ROS levels and ferroptosis marker protein levels in H/R-treated HL-1 cells and the myocardial tissue of I/R model mice, ultimately attenuating I/R-induced cardiac injury by inhibiting cardiomyocyte ferroptosis [115]. Gao et al. [116] confirmed that overexpression of lncRNA Gm47283 significantly increases Ptgs2 expression and the levels of ROS and MDA and inhibits GPX4 expression. They also demonstrated that a stem cell membrane-coated short interfering RNA (siRNA) targeting the lncRNA Gm47283 increased miR-706 expression in the advanced phase, suppressing Ptgs2 expression to reduce lipid peroxide toxicity and attenuating myocardial injury by inhibiting cardiomyocyte ferroptosis.

Patients with diabetes are vulnerable to a series of cardiovascular complications, including cardiomyocytes dysfunction and left ventricular longitudinal dysfunction. Ni and team found that inhibiting the lncRNA ZFAS1 attenuates diabetic cardiomyopathy (DCM) progression via sponging of miR-150-5p to activate cyclin D2 (CCND2), which leads to reduced collagen deposition and inhibiting of cardiomyocyte apoptosis and ferroptosis. Moreover, overexpression of miR-150-5p and CCND2 can improve cardiac function by significantly increasing the LVEF and LVFS and decreasing the LVEDD and LVESD [117]. Cardiac hypertrophy is a common maladaptive characteristic of multiple types of advanced CVDs, including coronary heart disease, valvular heart disease and myocarditis. An increasing number of studies have shown that lncRNAs can inhibit cardiac fibrosis and are potential biomarkers and novel therapeutic targets because they regulate the proliferation and transformation of cardiac fibroblasts [110, 118, 119]. Shi and colleagues found that forced expression of the lncRNA AABR07017145.1 induces MMP9/TIMP1 imbalance, which leads to ferroptosis of cardiac microvascular endothelial cells (CMECs) mediated by activation of TFR-1 and increased Fe^2+^ levels in a rat model of cardiac hypertrophy. However, miR-30b-5p, which was found to be significantly downregulated in a rat model and to target TIMP1, was shown to suppress the effect of the lncRNA AAB on ferroptosis. Additionally, further research demonstrated that silencing the lncRNA AAB and overexpressing miR-30b-5p are novel therapeutic strategies for cardiac hypertrophy [120].

Ferroptosis also plays a critical role in doxorubicin (DOX)-induced cardiomyopathy. Zhuang et al. [121] reported that DOX induces cardiomyocyte ferroptosis by activating the METL14/lncRNA KCNQ1OT1 signaling pathway and inhibiting miR-7-5p expression, which leads to an increase in TF levels, promoting iron absorption and lipid ROS production. Currently, cardiopulmonary bypass, coronary artery bypass grafting, cardiac valve replacement and heart transplantation are the main treatments for many complex CVDs, such as cardiac tumors. Cardiac injury during surgery is mainly associated with the stress response, hemodynamic instability and I/R-induced injury [122] and previous studies have shown that lncRNAs play functional roles in the injury process [123–125]. Overexpression of lncRNA SEMA5A-1T1 protects cardiomyocytes from H/R injury and against ferroptosis by sponging miR-143-3p to upregulate the expression of B-cell CLL/lymphoma 2 (BCL2) and SLC7A11 [126].

## CircRNAs that regulate ferroptosis in CVDs

As newly discovered critical ncRNAs, circRNAs, considered secondary products of canonical linear mRNA splicing, have been increasingly reported to participate in fundamental pathological and physiological processes in CVDs. Depending on their mode of biogenesis, circRNAs can be classified into three types, i.e., ciRNAs, ecRNAs and EIciRNAs [25, 26], and the biological functions of circRNAs have been gradually revealed. For example, circRNAs contain multiple binding sites for individual or multiple miRNAs to target other RNAs. They also interact with RNA-binding proteins and play roles in the assembly of scaffold components, splicing, gene transcription regulation and the translation of miRNAs into proteins or peptides.

In recent years, circRNAs have become a popular topic in biological research since they are highly conserved, show stable and specific expression in tissues and cells, can act as endogenous sponges to bind miRNAs and show regulatory capacity [131]. Through in-depth exploration of the molecular mechanisms underlying the effects of circRNAs on ferroptosis, researchers have confirmed their roles as potential molecular biomarkers and novel targets for the diagnosis and treatment of CVDs. For example, in an MI mouse model and hypoxia-treated cardiomyocytes, circRNA1615 and lipoprotein receptor-related protein 6 (LRP6) expression is significantly downregulated, while miR-152-3p expression is increased. A previous study showed that cardiac LRP6 is downregulated in patients with dilated cardiomyopathy, and cardiomyocyte-specific depletion of LRP6 causes lethal dilated cardiomyopathy, such as acute HF and cardiac dysfunction, resulting from the suppression of autophagy-mediated degradation and fatty acid utilization through Drp1/mTOR/TFEB signaling [127, 132]. Further research confirmed that siR-LRP6 increases the expression of the autophagy-regulated proteins LC3-A/B and ATG5 and decreases p62 expression. That is LRP6 plays a critical role in CVDs. Mechanistically, circRNA1615 sponges miR-152-3p to prevent LRP6-mediated autophagy-related ferroptosis in cardiomyopathy [127].

It has been reported that iron overload can impair heart function by increasing ROS levels via the Fenton reaction, which directly affects cardiac mitochondrial dynamics and promotes cardiac mitochondrial damage. Zheng et al. [128] found that the iron content in an HF mouse model was noticeably increased compared to that in the sham group, and decreased expression of GPX4 and increased expression of NOX1, ACSL4 and MDA indicated the occurrence of lipid peroxidation during HF. More importantly, FTH1 expression was markedly downregulated, and low circSnx12 expression and high miR-224-5p expression caused cardiac cell death by downregulating FTH1 and directly regulating iron overload in cardiomyocytes. However, in a mouse model of I/R, circ FEACR overexpression protected cardiomyocytes from I/R-induced ferroptosis by decreasing the levels of SLC7A11, GPX4, and MDA and lipid ROS production [129]. Mechanistically, FEACR directly binds to nicotinamide phosphoribosyltransferase (NAMPT) to increase NAMPT-dependent Sirtuin1 (Sirt1), which promotes the transcriptional activity of forkhead box protein O1 (FOXO1), which can upregulate FTH1, leading to the amelioration of MI and improvements cardiac function by inhibiting ferroptosis [129].

Maladaptive cardiac remodeling is an important determinant of morbidity and mortality that significantly affects life expectancy and quality of life. Knocking down circCmass1 was shown to decrease Ang II-induced neonatal cardiomyocyte hypertrophy in vitro and pressure overload-induced cardiac hypertrophy in TAC mice via the EIF4A3/TFR1 signaling axis to regulate ferroptosis [130] (Table 2). Notably, research on the role of ferroptosis-related circRNAs in CVDs is still lacking. Most of the recently published studies focus on cancer and cells [133]. Therefore, animal research clinical trials on CVDs are needed to ensure the safety and efficacy of the candidate molecules and methods.

## Conclusions and perspectives

CVDs represent a global health problem and have high rates of morbidity, mortality and disability. An understanding of how cardiomyocyte damage is involved in the pathology of CVDs and new viable therapeutic strategies are needed. On the role of ferroptosis in CVDs has attracted the attention of researchers. Recently accumulating evidence has revealed that various physiological processes and pathological stresses can trigger ferroptosis in humans and animals. Among these processes and stress, abnormal iron metabolism, amino acid metabolism, ROS production and excessive accumulation of lipid peroxides are critical ferroptosis inducers. However, research on ferroptosis is still in the initial stages, and many unanswered questions remain and need to be urgently answered.

First, there are multiple published studies on ferroptosis based on animal and cellular models, but experimental verification in vivo is lacking since it is difficult to accurately detect ferroptosis progression. However, recent studies have shown that we can detect morphological changes in ferroptotic cells at the subcellular level, including by analyzing plasma membranes, mitochondrial viscosity and the dynamic structure of nucleic acids during ferroptosis through probes, transmission electron microscopy and nucleic acid-responsive carbon dots [134]. And ferroptosis-related molecules, such as labile Fe (II), lipid peroxides, ROS, GSH and TFR1 can be detected through fluorescence probe, quantitative polymerase chain reaction, western blotting and flow cytometry [135, 136]. In clinical practice, positron emission tomography (PET) imaging can provide quantitative, three-dimensional images for in vivo assessment of labile Fe(II) ions levels [134], and cardiac magnetic resonance (CMR) combined with a processing workstation (Cvi42) can be used to analyze iron levels, cardiac structure and function, and the degree of fibrosis through the techniques of late gadolinium enhancement (LGE) and T1 and T2 mapping [137]. Moreover, the molecular mechanisms underlying ferroptosis and ferroptosis biomarkers remain to be identified. Clinical ferroptosis-related biomarkers for diagnosing CVDs and predicting the prognosis of CVDs patients have not yet been discovered. Finally, inhibitors of ferroptosis, such as Fer-1 and RSL3, have been proposed to effectively ameliorate cardiomyocyte injury, but their targets, the mechansims underlying their effects, their potential toxicity to untargeted organs and their feasibility for use in clinical settings remain unclear.

In recent years, ncRNAs have been shown to be important regulators of CVDs and to be involved in the life cycle of cardiomyocytes by affecting their differentiation, transcription and apoptosis rate. Additionally, the use of ncRNAs as biomarkers has increased due to their specificity, accessibility, stability and other distinguishing features. Specifically, ncRNAs crosslink ferroptosis and CVD markers, which helps in monitoring and interfering with ferroptosis-related CVDs. Through more in-depth research and the rapid development of bioinformatics technologies, the role of ferroptosis-associated ncRNAs in CVDs will be further elucidated in the future, and more databases will be established as integrated analysis platforms for researchers to study ferroptosis [138–140]. However, the role of ncRNA-mediated ferroptosis in CVDs and the mechanisms underlying pathophysiology of CVD remain to be further elucidated. Fewer ferroptosis-related studies have focused on lncRNAs and circRNAs than on miRNAs. In addition, cardiac fibrosis is a common pathological feature of various CVDs that results in cardiac systolic and diastolic dysfunction, conduction abnormalities and reduced patient compliance, ultimately leading to arrhythmias and HF. Sufficient evidence has indicated that ncRNAs are differentially expressed in myocardial fibrosis [141, 142], but further confirmation of these findings is need to identify the signaling pathways that ameliorate cardiac remodeling via ferroptosis and the potential roles of other pathways [93, 143]. Therefore, identifying ferroptosis-related ncRNAs involved in cardiac fibrosis may aid in preventing and treating of CVDs and predicting the prognosis of CVD patients.

In conclusion, both ferroptosis and ncRNAs play pivotal roles in the occurrence and development of numerous CVDs, but the underlying molecular mechanisms of ncRNA-induced ferroptosis in cardiomyocytes remain to be fully elucidated. As basic and clinical research on the molecular mechanisms of ferroptosis and interventions for inhibiting ferroptosis is being conducted, we believe that effective therapeutic strategies for CVDs are forthcoming.

## Data Availability

Not applicable.
